# Insights into growth retardation and dwarfism caused by goose parvovirus in goslings: a transcriptomic profiling study

**DOI:** 10.3389/fvets.2025.1529978

**Published:** 2025-05-06

**Authors:** Keshan Zhang, Guangliang Gao, Zhuping Chen, Hongyuan Zhang, Xianzhi Zhao, Qin Li, Lin Ma, Lecheng Wang, Yi Luo, Qigui Wang

**Affiliations:** ^1^Chongqing Engineering Research Center of Goose Genetic Improvement, Institute of Poultry Science, Chongqing Academy of Animal Science, Chongqing, China; ^2^College of Animal Science and Technology, Southwest University, Chongqing, China

**Keywords:** goose parvovirus, immunosuppression regulator factors, RNA-Seq, cell death, growth retardation, dwarfism

## Abstract

Goose parvovirus (GPV) poses a significant threat to the waterfowl industry as it results in a high mortality rate and stunted growth in surviving goslings, leading to significant economic losses. We used 120 goslings and goose embryo fibroblasts inoculated with the GPV SYG61 strain to study the pathogenesis of GPV by pathological and gene expression profile changes. Fourteen days after infection with the GPV SYG61 strain, goslings showed a mortality rate of 63.33%, along with dwarfism, significant weight loss, and severe histopathological lesions in the liver and jejunum. Serum analysis revealed a marked increase in the levels of immunosuppressive factors such as TGF-*β* and IL-10 (*p* < 0.01 or *p* < 0.05), while the levels of pro-inflammatory cytokines such as IL-4, IFN-*γ*, TNF-*α*, and IgG remained unaffected. In addition, GPV infection inhibited the proliferation of goose embryo fibroblasts and induced apoptosis, as demonstrated by transcriptomic analysis, which identified 285 differentially expressed genes (DEGs). These DEGs were enriched in pathways involved in the negative regulation of cell proliferation (GO: 0008285, 19/276, LogP = −12.62) and skeletal system development (GO: 0001501, 25/227, LogP = −12.51), with key genes including *IL6*, *CXCL8*, *PTGDS*, *PI15*, *MMP9*, *MMP13*, *MMP2*, *CCN3*, and *FAM180A*. Other DEGs were linked to the IL-17 signaling pathway (hsa04657) and the regulation of programmed cell death (GO: 0043068). Notably, GPV infection activated both apoptosis and ferroptosis through the upregulation of key regulatory genes such as *PTGS2*, *TF*, and *ASCL1* (*p* < 0.01). These findings indicated that GPV infection triggers inflammatory responses and programmed cell death, leading to high mortality in goslings, disturbs the expression of genes related to growth and skeletal development, and causes growth retardation and dwarfism in infected goslings. This study provides valuable insights into the pathogenic mechanisms of GPV and offers potential strategies to mitigate its impact and improve the productivity of the waterfowl industry.

## Introduction

Goose parvovirus (GPV) causes gosling plague, which is the most serious disease in geese farming, with a range of clinical symptoms such as dysentery, liver hemorrhage, intestinal embolism, viremia, delayed growth, and death in goslings (1, 2). The goose industry is an distinctive livestock industries in China. The meat geese output has reached approximately 515 million in 2023, accounting for 99% of Asia’s output and 94% of the world’s (3). However, the high mortality and growth retardation caused by GPV lead to significant economic losses in meat production. In addition, GPV infection can be latent in adult geese but vertically transmitted to the next generation, thus making its prevention and control difficult (4). The virus strain of GPV was first isolated by Dingyi Fang in 1961 from Yangzhou and was named SYG61 strain. The SYG61 strain was a standard virulent strain which was always used in laboratories to evaluate vaccine efficacy and immune protection or was attenuated to produce vaccine strains, such as SYG26-35 and SYG41-50. In 2015, a novel goose parvovirus (NGPV) that causes the short beak and dwarfism syndrome (SBDS) in waterfowl was detected in China, with typical clinical symptoms such as short beak, tongue protrusion, stubby tibia, lameness, and growth delay (5, 6). This NGPV detection shows that mutation and recombination in GPV have altered the clinical pathogenicity and enhanced the pathogenicity of impaired skeletal development, resulting in dwarfism.

The genomic structure and pathogenic mechanisms of parvovirus have been extensively investigated. It is well known that parvoviruses are the smallest and simplest single-stranded-DNA viruses that infect animals. They infect a broad range of species and can induce persistent, often subclinical infections, posing significant challenges to waterfowl farming and resulting in significant economic and health burdens (7). The parvovirus genome contains two open reading frames (ORFs) that encode the non-structural protein Rep and the capsid protein VP, which are further translated into functional proteins. Specifically, the Rep protein is cleaved into NS1 and NS2, while VP is cleaved into VP1, VP2, and VP3 (8).

NS1 plays a crucial role in viral DNA replication, host gene regulation, and apoptosis due to its ATPase and deconjugase activities (9, 10). NS2 synergizes with NS1 to promote the synthesis of viral nucleic acids and proteins (11). Yan et al. showed that the goose parvovirus NS1 increases the expression of apoptosis-inducing factor (AIF) and reactive oxygen species (ROS), which activates the AIF–mitochondrial pathway and induces apoptosis in goose embryo fibroblast (GEF) cells (12). This suggests that NS1 directly triggers host cell death, potentially promoting viral spread and persistence. Similarly, Xu et al. found that the C-terminal transactivation domain (TAD2) of the NS1 protein from parvovirus B19 induces cell cycle arrest at the late S phase and G2 phase by activating the ATR–CHK1–CDC25C–CDK1 pathway while also triggering a DNA damage response (DDR) to facilitate viral DNA replication in human erythroid progenitor cells (13). These findings highlight the multifunctional role of NS1 in manipulating host cellular processes and promoting viral replication. In addition, canine parvovirus (CPV) NS1 induces apoptosis in HeLa cells through ROS accumulation and caspase activation, which demonstrates the conserved pro-apoptotic function of NS1 across parvovirus species (14).

The capsid protein VP plays a key role in viral virulence and pathogenicity, elicits specific antibody responses, and contributes to immune evasion (15). Yang et al. reported that the NGPV VP1 protein interacts with IRF7, blocks the type I interferon signaling pathway, and helps the virus evade the immune system (16). This immune evasion strategy allows the virus to establish persistent infections, which—though often subclinical—can have long-term impacts on host health. Mutations in the capsid protein of CPV affect its binding to transferrin receptor type 1 (TfR1), thereby modifying viral infectivity and host range (17). VP proteins are also involved in immunosuppression, which can lead to long-term latent infections. For instance, bovine parvovirus VP1 inhibits IFN-*β* production via the RIG-I-like receptor pathway in bovine turbinate cells, allowing the virus to evade immune detection (18). CPV also modifies capsid receptor and antibody binding sites, leading to host range switch and neutralization escape (19). Although previous studies have shown that parvoviruses induce apoptosis in host cells, the specific mechanisms by which a GPV infection alters host gene expression to activate immune and inflammatory responses, induce apoptosis, and inhibit bone development are not well understood.

To determine the changes induced by a viral infection that are involved in growth retardation and viral persistence in the host, we investigated the effects of GPV SYG61 strain infection in goslings and GEF cells. We analyzed the impact of SYG61 infection on body weight, serum inflammatory factor levels, histopathological changes, and proliferation and apoptosis of GEF cells. Furthermore, we carried out transcriptomic analysis to identify virus-induced changes in host gene expression. Our findings provide valuable insights into the mechanisms underlying GPV-induced growth retardation and latent infection and contribute to a better understanding of how viral factors and host responses interact to facilitate persistent infection and developmental abnormalities in geese.

## Materials and methods

### Animals and virus strains

A total of 120 four-day-old healthy Sichuan white goslings and 10-day-old goose embryos were acquired from the breeding geese kept in isolation facilities in Chongqing Anfu Poultry Research Base; all individual goslings were free of GPV and other pathogens (AI, GMPV, CoCV, AsTV, AR, etc.). A standard highly virulent GPV SYG61 strain maintained at the Poultry Research Institute of Chongqing Academy of Animal Sciences was used in this study. This SYG61 strain had a strong pathogenicity of 10^6.1^ELD_50_ /0.2 mL.

### Challenge trial

The goslings were randomly allocated into two groups (infected group and control group, 60 goslings per group), housed in four isolators (30 goslings per isolator) in a BSL-2 laboratory, and fed the same commercial broodstock. The infected group was intramuscularly inoculated with 0.1 mL of GPV SYG61 allantoic fluid, while the control group received an equivalent dose of sterile saline. On days 3, 7, and 14 postinfection, eight goslings from each group were randomly selected for blood collection via the jugular vein. The serum samples were sent to Shanghai Hengyuan Biotechnology, Co., for the analysis of inflammatory factors [interleukin (IL)-4, IL-6, IL-8, IL-10, INF-*γ*, TGF-*β*, TNF-*α*, and IgG], and the body weight of each group was measured 14 days after infection. Tissues from the liver, spleen, jejunum, ileum, and other organs were collected from diseased and deceased goslings, fixed in 10% formalin, subjected to paraffin embedding, and stained with hematoxylin and eosin (HE). Six goslings from the control group were also randomly selected and subjected to autopsy, and the corresponding tissues were collected and processed similarly.

### Isolation, cultivation, and infection of goose fibroblasts

GEF cells were isolated from 10-day-old goose embryos following the conventional method. Cell density was determined, and the cells were inoculated on 12- well and 96-well cell plates, incubated with DMEM/F12 medium containing 10% fetal bovine serum (FBS), and cultured at 38°C and 5% CO_2_. When the cell confluence reached 70%, they were inoculated with 10^6^ copies and 10^5^ copies GPV virus particles/well for 1 h [quantified by TaqmanPCR; the standard curve: y = −3.0664x + 39.395; R^2^ = 0.9901; primer sequences: F-5’-GTTCCTTTCCACAGCATGTTC-3′, R-5’-CTGCTGCTGTCTACCTCATT-3′, P-5′-(FAM) CAGCCTGTCTAAGTCCTGTGAATGAG C (Eclipse)-3′]. Then, the medium was replaced with 5% FBS maintenance medium. The cells were monitored for cytopathic effects and harvested 48 h after infection for transcriptomic analysis, with five replicates per treatment.

### Cell proliferation and apoptosis assay

Goose fibroblasts were plated on 96-well plates (5 × 10^4^ cells/well) with DMEM/F12 medium containing 10% FBS and cultured at 38°C and 5% CO_2_. Three wells were infected with the SYG61 strain at 70% confluence, and three wells received saline and served as controls. Cell proliferation was assessed 24 h after infection using the EdU assay (RIBOBio Co, Guangzhou, China). The cells were incubated with 50 μM EdU for 2 h, fixed with 4% paraformaldehyde, stained with Apollo, and visualized under a fluorescence microscope (EVOS M5000, ThermoFisher, United States). EdU-positive cells were quantified in at least three separate fields. Apoptosis of the infected cells was measured 48 h after infection using the TUNEL assay (R11059.2, RIBOBio Co, Guangzhou, China), following fixation with 4% paraformaldehyde and permeabilization with 0.5% Triton-100. The cells were subjected to TdT enzyme reaction, followed by Apollo and DNA staining, and were observed under the fluorescence microscope.

### Transcriptomic sequencing analysis of goose embryo fibroblasts infected with GPV

Total RNA was extracted from two groups of fibroblasts (five infected wells and five control wells) using Trizol reagent (Invitrogen Life Technologies, Carlsbad, CA, United States). RNA quality was assessed using RIN values according to the manufacturer’s instructions. cDNA libraries of the 10 samples were constructed using Illumina NEBNext^®^ Ultra™ RNA Library Prep Kits (New England Biolabs, NEB, Ipswich, MA, United States) according to the manufacturer’s instructions. Subsequently, cDNA libraries were purified, evaluated, and sequenced using the (Illumina, San Diego, CA, USA), (Beckman Coulter Inc., Brea, CA, USA) (Agilent Technologies, Santa Clara, USA) 2,100 system, and Illumina platform, respectively. After removing the adaptors, low-quality sequences, sequences with uncertain bases, and sequences less than 50 bp, the reads were mapped to the Tianfu meat goose genome using STAR 2.7.10b and were assembled Cufflinks (http://cufflinks.cbcb.umd.edu/) (20, 21). Significant DEGs were screened (q value< 0.05, fold change ≥2, and false discovery rate <0.01) and subjected to GO and KEGG pathway enrichment analysis on the Metascape web server.^1^ Protein–protein interaction (PPI) network analysis was carried out for the top 50 DEGs on the STRING database.^2^

### RT-qPCR for the selected candidate DEGs

To validate DEGs from the transcriptomic analysis, the RNA obtained after transcriptomic sequencing was transcribed into cDNA using GoScript™ Reverse Transcriptase (A5001, Promega, United States), and qPCR was performed using LightCycler96 (Roche, Swiss) with the GoTaq^®^ qPCR Master Mix (A6001, Promega, United States). *GAPDH* was selected as the endogenous gene. The primers of the candidate genes are presented in Supplementary Table S1. The 2^−ΔΔCt^ method was used to calculate the relative mRNA expression level of the candidate genes. All analyses and figures were reproduced using (OriginLab, Northampton, MA, USA).

### Statistical analysis

All statistical analyses were conducted using IBM SPSS Statistics (v. 19.0 IBM Corp., Armonk, NY, United States). The results were expressed as mean ± standard error of the mean (SEM), and *p* values of <0.05, <0.01, and <0.001 were considered significant, very significant, and extremely significant, respectively. One-way ANOVA and Student’s *t* test were used to determine whether the means of the two treatment groups were significantly different.

## Results

### GPV infection induces high mortality and stunted growth in goslings

To determine the pathogenic effects of the highly virulent SYG61 strain of GPV, we challenged 60 four-day-old Sichuan white goslings with the virus via intramuscular injection and compared the effects with a control group of 60 goslings. Within 3 days of injection, significant clinical symptoms developed in infected goslings, including depression, diarrhea, and chilling. The SYG61 strain resulted in the death of 38 goslings, with a mortality rate of 63.33% in the infected group. Fourteen days after infection, the average body weight of the surviving goslings was significantly reduced compared with the control group (445.51 ± 54.03 g vs. 606.79 ± 51.26 g, *p* < 0.01; Table 1). Notably, 30 days postinfection, four goslings had a body weight of less than 800 g and showed visible signs of dwarfism, while the average body weight of the remaining surviving goslings and the control group was 1,398 ± 72.56 g and 1,654 ± 60.44 g, respectively. These findings indicate that GPV infection results in severe stunted growth and high mortality in goslings.

**Table 1:** Effect of GPV infection on mortality rate, body weight, and cytokines of goslings.

Groups	Mortality rate	18-day body weight (g)	34-day body weight (g)	TGF-β (ng/L)	IgG (ng/L)	TNF-α (ng/L)	INF-γ (ng/L)	IL-4 (ng/L)	IL-6 (ng/L)	IL-8 (ng/L)	IL-10 (ng/L)
Infected group	0.63	445.51 ± 54.03^A^	1,398 ± 72.56 g ^a^	337.89 ± 14.32^A^	91.06 ± 6.06	287.14 ± 17.36	93.42 ± 6.53	141.8 ± 10.11	47.83 ± 3.63	91.15 ± 5.26^A^	73.21 ± 6.41^a^
Control group	0.00	606.79 ± 51.26^B^	1,654 ± 60.44gb	322.90 ± 14.95^B^	90.94 ± 8.82	292.30 ± 17.4	92.01 ± 7.38	139.23 ± 10.93	47.26 ± 4.17	82.90 ± 6.37^B^	68.16 ± 4.49^b^

Each treatment group has 60 goslings. During the final weight measurement, only 22 goslings remained in the infected group and 38 died. Each treatment group had eight copy samples in the serum cytokines level assay. Values with the same superscript letter show differences that are not significant (*p* > 0.05); those with different lowercase superscript letters show significant differences (*p* < 0.05); those with different uppercase superscript letters show extremely significant differences (*p* < 0.01).

### GPV infection increases serum concentrations of immunosuppressive factors in goslings

Fourteen days after GPV infection, we measured serum concentrations of TGF-*β*, IgG, TNF-*α*, INF-*γ*, IL-4, IL-6, IL-8, and IL-10 using ELISA. As shown in Table 1, the average concentrations of TGF-β and IL-10 were significantly increased in the infected group (*p* < 0.01 or *p* < 0.05). Notably, the concentration of inflammatory cytokine IL-8 was also significantly increased (*p* < 0.01) in the infected group, with an average concentration of 73.21 ng/L. In contrast, there were no significant differences in serum levels of pro-inflammatory cytokines TNF-*α*, INF-*γ*, and IL-4 between the infected and control groups. Similarly, as expected, IgG concentrations remained unchanged. TGF-*β*, a critical immunosuppressive regulator, inhibits the proliferation of T-lymphocyte subpopulations and B lymphocytes while suppressing IgG, IgM, and TNF-*α* production (22). IL-10, a cytokine synthesis inhibitory factor, also suppresses the production of various inflammatory cytokines, including IFN-*γ*, TNF-α, and IL-6 (23, 24). These findings indicate that GPV infection significantly increases the serum levels of immunosuppressive factors, thereby impairing the antiviral response in goslings.

### Clinical and histopathological findings following SYG61 strain infection

**Figure 1:**
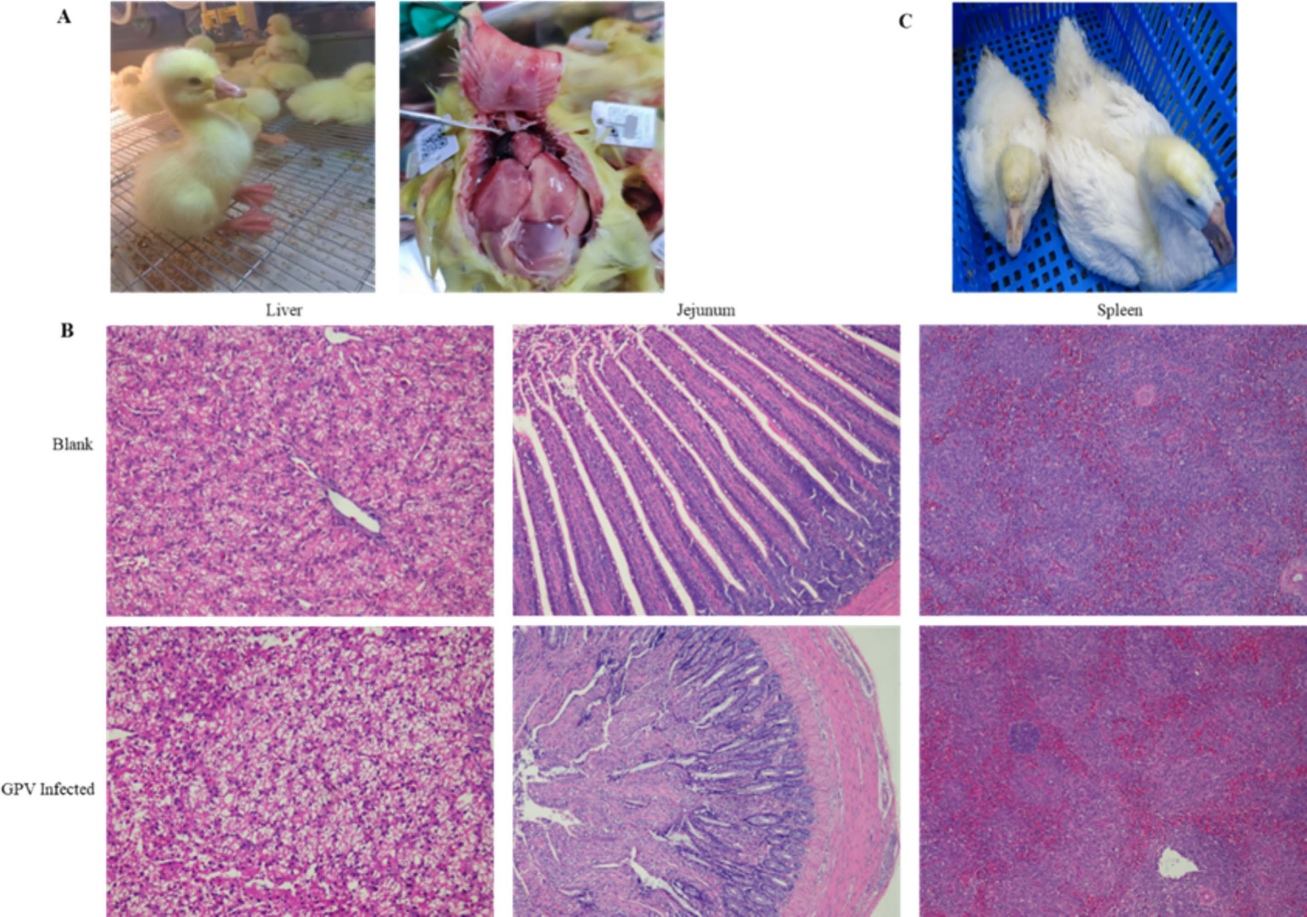
Clinical and histopathological observations in goslings infected with GPV. **(A)** SYG61-infected goslings showed depression, diarrhea, and death. **(B)** Results of the histopathological examination of the liver, spleen, and jejunum of dead goslings. Sludge and steatotic lesions were observed in the liver. Mucosal epithelium, degenerated and detached, was observed in the jejunum, the number of vacuolated goblet cells increased, the intestinal villi shortened and disintegrated, and the inflammatory cells infiltrated. The splenic red pulps were filled with a large number of erythrocytes, and the number of white pulp microsomes increased. **(C)** Several postinfection surviving goslings showed dwarfism.

Three days after the SYG61 strain injection, the infected goslings showed pronounced clinical symptoms, including depression, a fluffy coat, diarrhea, and chilling. Autopsy of the deceased goslings revealed marked intestinal enlargement, thin-walled structures, and accumulation of yellowish mucus (Figure 1A). Histopathological examination of liver, spleen, and jejunum tissues from all deceased goslings indicated significant lesions. In the liver of the infected goslings, sludge and steatotic lesions were prevalent. Examination of the jejunum revealed degeneration and detachment of the mucosal epithelium, an increase in the number of vacuolated goblet cells, shortened and disintegrated intestinal villi, and notable inflammatory cell infiltration. In contrast, no microscopic lesions were detected in the control group (Figure 1B). The replication of parvovirus depends on the host cell DNA replication machinery, which predominantly targets mitotically active tissues such as the intestine, reproductive tract, and erythrocytes, which elucidates that the severe intestinal lesions and resultant sepsis are due to GPV infection. Notably, 30 days after infection, several goslings in the infected group showed signs of dwarfism (Figure 1C).

### Effects of GPV infection on cell proliferation and apoptosis in goose embryo fibroblasts

**Figure 2:**
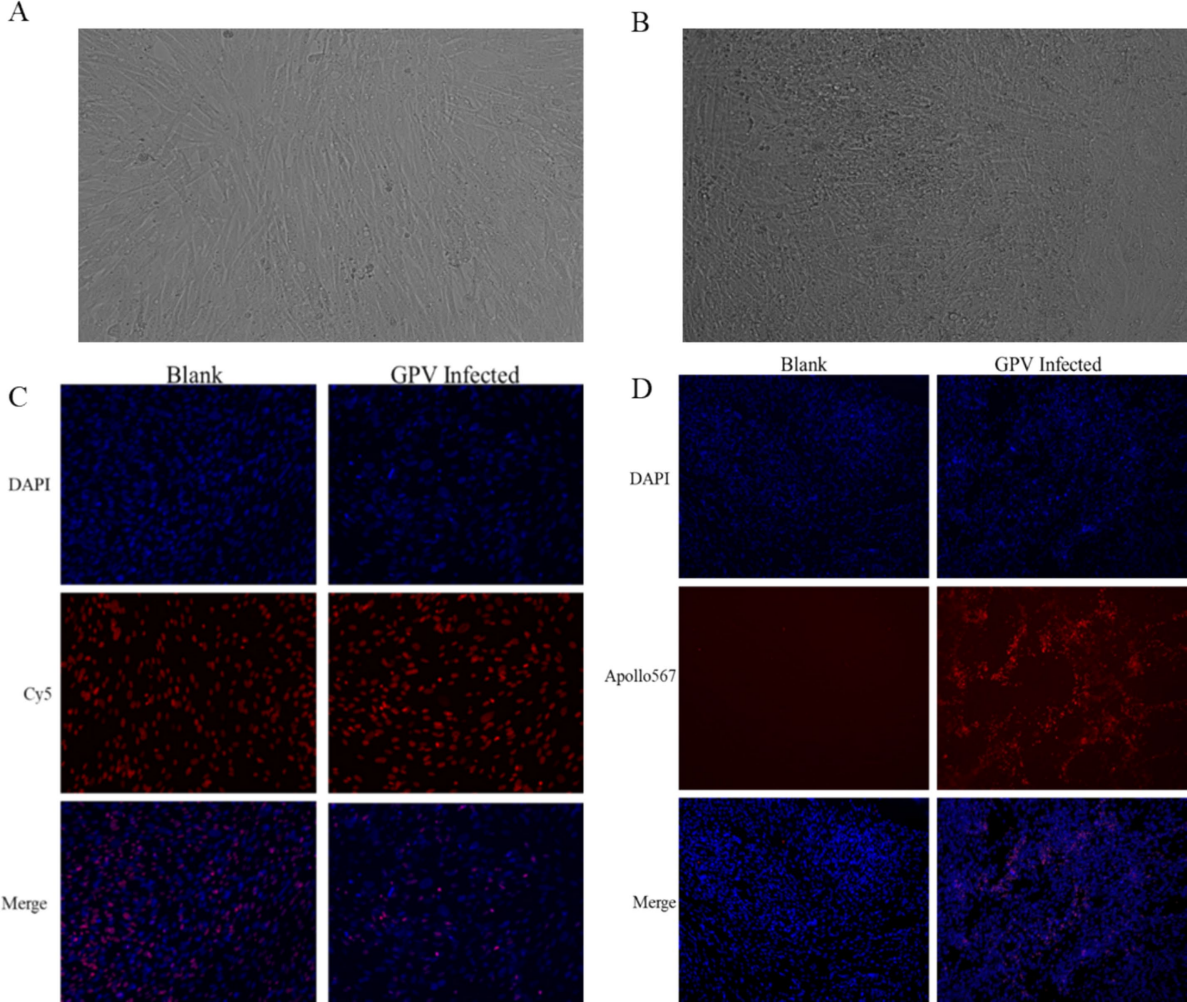
Effects of GPV infection on GEFs. **(A)** Normal goose fibroblasts were long and pike-shaped and neatly arranged. **(B)** The GPV-infected cells became rounded, aggregated into clusters 48 h after infection. **(C)** The number of EdU-positive cells was decreased in the GPV-infected group 24 h after infection. **(D)** The apoptotic signal was increased significantly in the GPV-infected group 48 h after infection.

The infection of GEF cells with the SYG61 strain resulted in characteristic cytopathic effects. Under normal conditions, GEF cells showed a long, spindle-shaped morphology and were neatly arranged. However, after GPV infection, they underwent morphological changes, becoming rounded, aggregating into clusters, and exhibiting fusion with indistinct borders and enhanced refractoriness (Figures 2A,B). In addition, cell proliferation assays indicated a significant reduction in the number of EdU-positive fibroblasts in the infected group compared with the control group, demonstrating the inhibition of cell proliferation (Figure 2C). Furthermore, apoptotic signals were clearly evident in the infected cells, which appeared to be detaching, whereas they were scarcely detectable in the control group (Figure 2D).

### Effects of GPV infection on transcriptome in goose embryo fibroblasts

**Figure 3:**
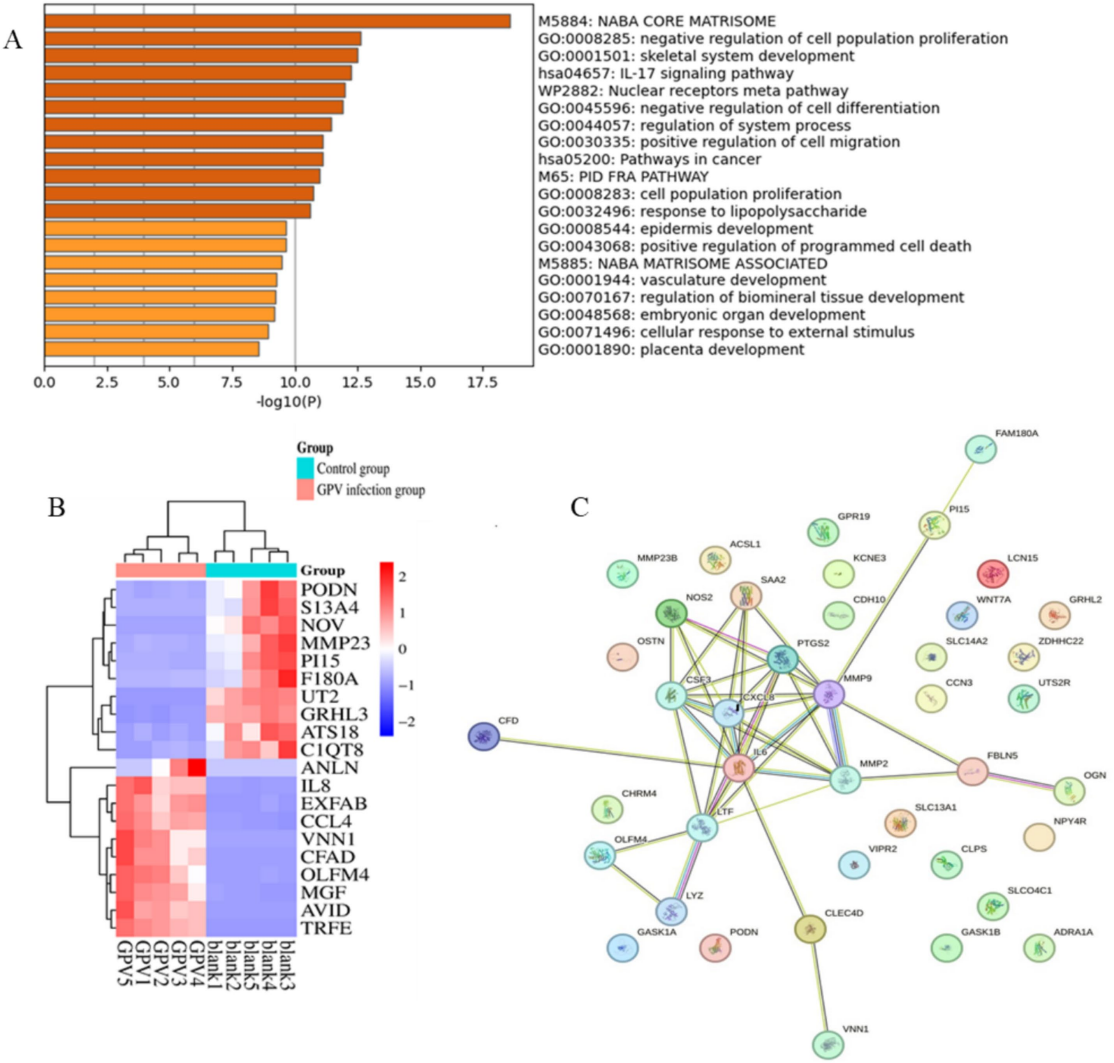
Functional enrichment and protein–protein interaction analysis of DEGs caused by GPV infection on GEFs. **(A)** The top 20 enriched pathways were listed. *p-*value cutoff at 0.01, and minimum enrichment at 1.5. **(B)** Heatmap shows the expression of the top 20 DEGs between control and GPV-infected groups. **(C)** The protein–protein interaction results of the top 50 DEGs. PPI enrichment *p*-value < 1.0e^−16^.

The size of the transcriptomic data was 8.09–12.71Gb, and clean reads were mapped to the goose reference genome. The total mapped rate was higher than 88.5% for each sample (Table 2). Analysis of the transcriptomic data revealed that GEF cells produced 285 significant DEGs (∣log2FC∣ ≥ 1, FDR ≤ 0.01) after GPV infection, of which 176 were significantly downregulated and 109 were significantly upregulated. These DEGs were primarily enriched in the negative regulation of cell population proliferation (GO: 0008285, logP = −12.62), skeletal system development (GO: 0001501, logP = −12.51), and IL-17 signaling pathway (hsa04657, logP = −12.25); positive regulation of cell migration (GO: 0030335, logP = −11.11) and programmed cell death (GO: 0043068, logP = −9.64); and cellular response to external stimulus (GO: 0071496, logP = −8.94; Figures 3A,B). The downregulated DEGs were primarily enriched in skeletal system development (GO: 0001501, logP = −29.61), growth (GO: 0040007, logP = −12.77), and bone development (GO: 0060348, logP = −9.36). However, the most highly enriched GO terms of upregulated DEGs that were related to inflammation included the IL-17 signaling pathway (hsa04657, logP = −11.50), cellular response to cytokine stimulus (GO: 0071345, logP = −11.47), and regulation of cellular response to lipids (GO: 0071396, logP = −12.25; Supplementary Figure S1). The DEGs were subjected to PPI network analysis. As shown in Figure 3C, the close biological interactions of candidate DEGs, including C-X-C motif chemokine ligand 8 (*CXCL*8), transferrin (*TF*), prostaglandin-endoperoxide synthase 2 (*PTGS*2), matrix metallopeptidase 9 (*MMP*9), *MMP*2, *IL6*, family with sequence similarity 180 member A (*FAM*180*A*), and peptidase inhibitor 15 (*PI1*5), showed convergent pathways, including skeletal system development, cell proliferation, and inflammation. These findings reveal novel insights into the theory that GPV infection impairs skeletal development, negatively regulates cell proliferation, and accelerates programmed cell death and immunosuppression.

**Table 2:** Characteristics of reads from the transcriptome of goose embryonic fibroblast cells with different treatments.

Samples	Paired reads	Data size(Gb)	Unique reads	Uniquely mapped (%)	Multi-reads	Multi-mapped (%)	Total mapped (%)
Blank1	33,581,015	10.0743045	28,926,625	86.14	1,083,847	3.23	89.37
Blank2	28,044,066	8.4132198	24,782,909	88.37	836,753	2.98	91.35
Blank3	26,967,596	8.0902788	24,062,042	89.23	780,480	2.89	92.12
Blank4	34,837,440	10.451232	30,827,635	88.49	1,018,572	2.92	91.41
Blank5	42,393,288	12.7179864	37,166,876	87.67	1,264,596	2.98	90.65
GPV1	39,982,698	11.9948094	34,322,378	85.84	1,373,462	3.44	89.28
GPV2	37,327,658	11.1982974	32,234,697	86.36	1,257,212	3.37	89.73
GPV3	39,561,102	11.8683306	33,813,599	85.47	1,418,103	3.59	89.06
GPV4	34,059,710	10.2179113	28,878,563	84.79	1,263,752	3.71	88.5
GPV5	31,371,602	9.4114806	26,765,695	85.32	1,161,743	3.71	89.03

Blank1–5 represent the fibroblast cell treatment with saline; GPV1–5 represent the fibroblast cell treatment with 10^5^ copies of the GPV SYG61 strain.

### Validation of DEGs via RT-PCR analysis

**Figure 4:**
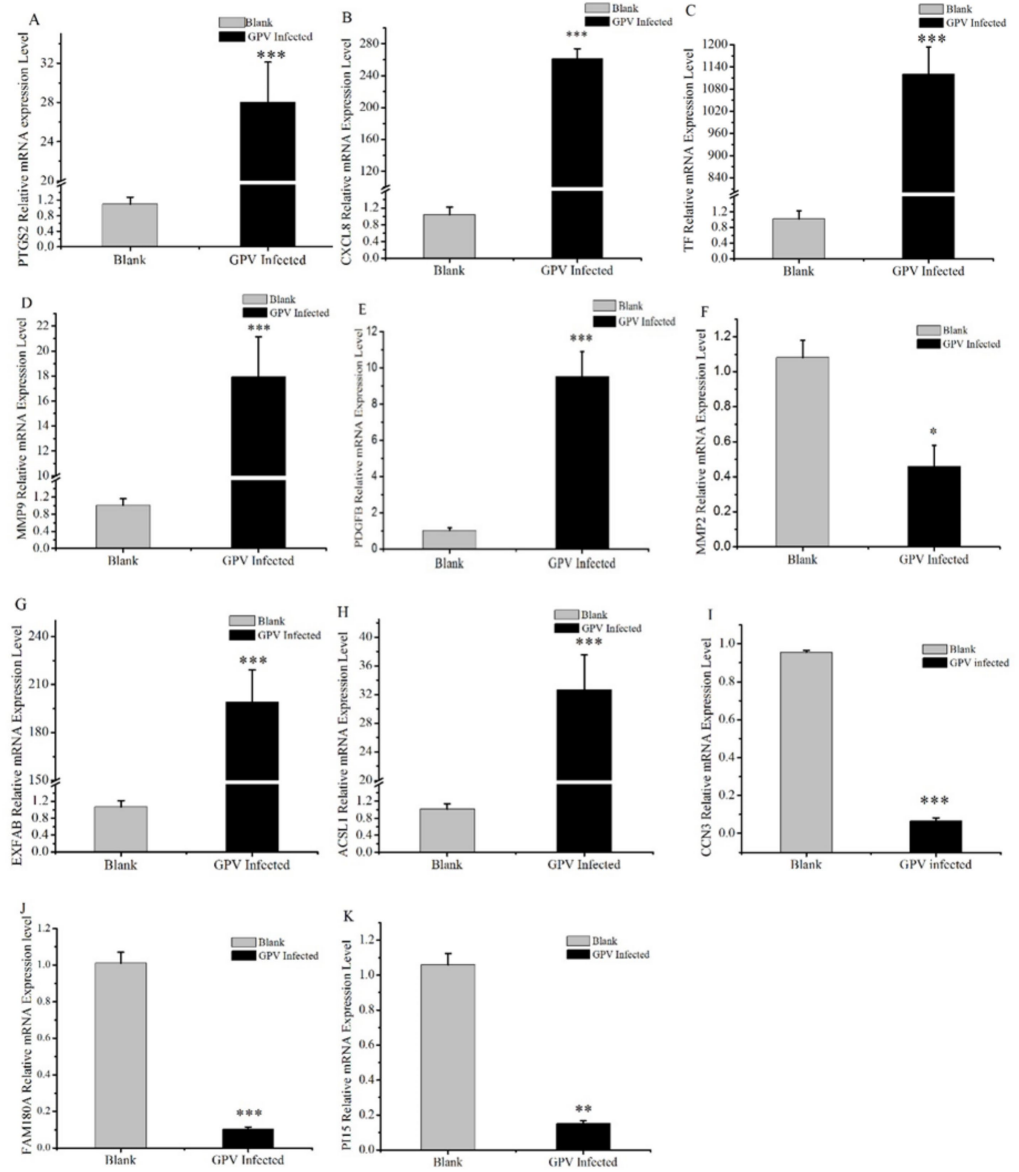
Validation results of DEGs selected by RT-qPCR. Some candidate DEGs that are involved in skeletal development, negative regulation of cell proliferation, and cell death acceleration were selected and validated by RT-qPCR. *represents that the difference was significant at *p* < 0.05, **represents that the difference was significant at *p* < 0.01, and ***represents that the difference was significant at *p* < 0.001.

To validate the candidate DEGs associated with GPV infection, we conducted an RT-PCR analysis on *CXCL*8, *LTF*, *PTGS*2, *MMP*9, *MMP*2, *PGDFB*, *EXFABP*, acyl-CoA synthetase long-chain family member 1 (*ACSL*1), *CCN*3, *FAM*180*A*, and *PI1*5. It showed that compared with the control group, the mRNA levels of *CXCL8*, *TF*, *PTGS2*, *MMP9*, *PGDFB*, *EXFABP*, and *ACSL1* were significantly increased in response to GPV infection with extremely significant upregulation (*p* < 0.001), whereas the mRNA levels of *MMP2*, *CCN3*, *FAM180A*, and *PI15* were significantly or extremely significantly downregulated (*p* < 0.05 or *p* < 0.01, respectively). Notably, the relative mRNA level of transferrin (*LTF*) in infected fibroblasts increased more than 1,000-fold (Figure 4C), thus reinforcing the transcriptomic data and providing strong evidence for the role of these DEGs in the host response to GPV infection.

## Discussion

GPV is a small single-stranded-DNA virus with a 5.1-kb genome that is incapable of independent replication and relies on the host cell DNA replication machinery, which makes it more likely to infect mitotically active cells such as intestinal epithelial cells, germinal epithelium, and erythrocytes (25). This explains why GPV infection is more severe in goslings, which have a high number of dividing cells, while causing subclinical infections in adults (26). In the present study, the SYG61 stain resulted in a mortality rate of up to 63.33% in goslings and significantly suppressed weight gain and growth. SYG61-infected goslings had significantly lower body weight compared with controls (*p* < 0.01, Table 1). As widely reported, NGPV causes SBDS in geese. Some SYG61-infected geese also showed signs of dwarfism (Figure 1C).

The transcriptomic analysis provided insights into the mechanisms underlying dwarfism after GPV infection. DEGs were enriched in pathways related to the negative regulation of cell proliferation (GO: 0001501, 19/276, Log *p* = −12.62) and skeletal system development (GO:0008285, 25/227, Log *p* = −12.51), including *IL6*, *CXCL8*, *PTGDS*, *PTGS2*, *LTF*, *MGP*, *MMP*9, *MMP*13, *CCN*3, *OGN*, *TNFRSF11B*, *SFRP2*, TBX15, *PI*15, *FAM180A*, and *GPR68*. Previous studies have shown that parvovirus B19 (B19V) significantly increases MMP9 activity and enhances the expression of TGF-*β*, NF-κB, IL-6, TNF-*α*, and IL-1β in skin fibrosis of systemic sclerosis (27). The NS1 of B19V acts as a trans-acting transcriptional activator on the NF-κB site of the IL-6 promoter region, which can induce *IL*-6 production (28). Consistent with these findings, our results showed that GPV infection also increases MMP9 and IL-6 mRNA expression and serum TGF-β levels in the infected group.

*IL-6* plays a role in inflammation and B-cell maturation and has also been shown to be crucial in skeletal development. In IL-6-overexpressed transgenic mice, osteopenia, severe alterations in bone microarchitecture, increased osteoclastogenesis, and reduced osteoblast activity were observed (29). *MMP*9 is involved in bone resorption and regulates citric acid metabolism during the osteogenic differentiation of bone marrow mesenchymal stem cells. Increased serum levels of MMP9 are an indicator of decreased bone density and osteoporosis (30, 31). *CXCL8/IL8* functions as a major chemoattractant to neutrophils, which can induce inflammation and sustain the body in the inflammatory state (32). *CCN*3 is associated with thinness due to insulin resistance and is known to negatively regulate bone regeneration and maintain bone density (33). *PI15* regulates cell proliferation and growth and reverses skeletal muscle atrophy (34). Although the function of FAM180A is not well understood, it has been shown to be downregulated by TGF-*β* signaling, potentially playing a role in tissue regeneration (35). In our study, we observed increased serum TGF-*β* levels and downregulated expression of FAM180A. In addition, serum levels of TGF-β, IL-8, and IL-10 were significantly increased in the infected group (Table 1). TGF-β, an effector cytokine, is involved in immunosuppressive functions and regulates Treg and Th17 cell activity, as well as inhibiting stimulated T-cell proliferation (22, 36). Our results showed significantly reduced proliferation of fibroblasts infected with GPV (Figure 2C). The significant upregulation of CXCL8, IL-6, and MMP9, along with the downregulation of CCN3, PI15, and FAM180A, suggests that the impaired skeletal development and negative regulation of cell proliferation are key contributors to dwarfism in GPV-infected goslings. Furthermore, high serum levels of TGF-β and IL-10, along with low levels of IgG and pro-inflammatory cytokines (TNF-*α*, IFN-*γ*, and IL-4), may contribute to immunosuppression and persistent infection.

GPV infection was also found to induce apoptosis in GEF cells, as evidenced by pronounced apoptotic signals in the infected cells compared with controls (Figure 2D). Previous studies have shown that the NS1 protein of parvoviruses is involved in viral replication, induction of cell cycle arrest, DDR, and ultimately cell apoptosis or necrosis (26, 37). Cyclins and cyclin-dependent kinases (CDKs) play a crucial role in controlling the cell cycle. The NS1 protein of human parvovirus B19V significantly increases CDC2-cyclin B1 kinase activity by phosphorylating cyclin A, cyclin B1, and CDC2 during viral replication (13, 38). The NS1 protein of minute virus of mice was found to be associated with CDK inhibitors p21CIP1, cyclin A, CDK, cyclin E, and CDK2, resulting in S-phase and G2-phase cell cycle arrest (39, 40). Meanwhile, multiple mechanisms have been proposed for parvovirus-induced apoptosis, including increased expression of pro-apoptotic genes p53, Bax, and Bad, as well as activation of caspase-3 and caspase-9. Transfecting the NS1 of B19V into human erythroid cell lines has been shown to increase the expression levels of apoptosis-related genes *p*53, *Bax*, and *Bad* and activate caspase-3 and caspase-9 (41). The *NS1* of CPV2 has been shown to increase the BAX/Bcl2 ratio, initiate the destruction of the mitochondrial membrane potential, and trigger the aggregation of ROS (42, 43).

According to our transcriptomic data, several important DEGs were enriched in the positive regulation of programmed cell death (GO: 0043068, Log *p* = −9.64), such as *FASLG*, *PTGS2*, *MMP9*, *IL6*, *Bcl2L11*, *ATF4*, *HMOX1*, *HOXA5*, *HTRA1*, *SFRP2*, *SLC9A1*, and *VNN*. FASLG binds to FAS and initiates apoptosis through the FAS/FASLG signaling pathway, which plays a key role in immune regulation, including cytotoxic T-cell-induced cell death and activation-induced cell death of T cells (44, 45). *Bcl2L11* is a member of the BCL-2 protein family and functions as an apoptotic and ferroptosis activator (46, 47). Notably, GPV infection also alters transcript levels of some genes associated with ferroptosis. Ferroptosis is a non-apoptotic form of programmed cell death, which is mainly related to iron metabolism disorder, imbalance in the amino acid antioxidant system, and lipid peroxide accumulation (48). X.Q. et al. reported that ferroptosis-related upregulation of PTGS2, a marker for lipid peroxidation, is associated with inflammation and encephalitis caused by HSV-1 (49). In our study, *PTGS2* was significantly upregulated in GPV-infected fibroblasts, as was TF, with more than 1,000-fold upregulation. TF transports iron into the cytoplasm, where it can drive the Fenton reaction, generating large amounts of ROS that lead to lipid peroxidation and ferroptosis (50). In addition, another ferroptosis promoter ACSL1 was also significantly upregulated in the GPV-infected group. ACSL1 incorporates *α*-eleostearic acid into specific lipid species, including DAGs and TAGs, thus forming a mass of lipid droplets and covalent lipid droplet polymers and triggering lipid peroxidation and ferroptosis (51). We also observed lipid droplet accumulation in the liver of GPV-infected goslings (Figure 1B). Overall, GPV infection of GEF cells upregulates genes associated with apoptosis and ferroptosis, leading to cell death and lysis. These findings underscore the multifaceted impact of GPV on host cellular mechanisms, which results in high mortality rates in infected goslings.

In conclusion, our findings elucidate the detrimental effects of GPV infection in goslings and GEF cells, highlighting the virus’s role in inducing high mortality, growth retardation, and dwarfism. Furthermore, this study provides insights into the underlying mechanisms of these clinical symptoms, demonstrating that GPV infection activates apoptosis and ferroptosis, inhibits cell proliferation, disrupts skeletal development, and induces persistent immunosuppression. These findings enhance our understanding of the pathogenic mechanisms of GPV and interaction networks between GPV and its host.

## Data Availability

The datasets presented in this study can be found in online repositories. The names of the repository/repositories and accession number(s) can be found at: https://www.ncbi.nlm.nih.gov/, PRJNA1175047.

## Footnotes

^1^ http://metascape.org/

^2^ https://cn.string-db.org/

## References

[1] Liu M Li L Zhang W Wang L Cui Y Hao X. Bone lesions and intestinal barrier disruption caused by the isolated novel goose parvovirus infection in ducks. Microb Pathog. (2024) 194:106825. doi: 10.1016/j.micpath.2024.106825, PMID: 39074517

[2] Xiao S Chen S Cheng X Lin F Wang S Zhu X. The newly emerging duck-origin goose parvovirus in China exhibits a wide range of pathogenicity to main domesticated waterfowl. Vet Microbiol. (2017) 203:252–6. doi: 10.1016/j.vetmic.2017.03.012, PMID: 28619152

[3] Hou S Liu L. Waterfowl industry and technology development report 2023. Chinese J Animal Sci. (2024) 60:4. doi: 10.19556/j.0258-7033.20240202-11

[4] Chen H Tang Y Dou Y Zheng X Diao Y. Evidence for vertical transmission of novel duck-origin goose parvovirus-related parvovirus. Transbound Emerg Dis. (2016) 63:243–7. doi: 10.1111/tbed.12487, PMID: 26890433

[5] Chen H Dou Y Tang Y Zhang Z Zheng X Niu X. Isolation and genomic characterization of a duck-origin GPV-related parvovirus from Cherry Valley ducklings in China. PLoS One. (2015) 10:e0140284. doi: 10.1371/journal.pone.0140284, PMID: 26465143 PMC4605506

[6] Wang J Wang Y Li Y Gao Y Li Y Jiang Z. Reproduction and pathogenesis of short beak and dwarfish syndrome in Cherry Valley Pekin ducks infected with the rescued novel goose parvovirus. Virulence. (2022) 13:844–58. doi: 10.1080/21505594.2022.2071184, PMID: 35481463 PMC9090291

[7] Kailasan S Agbandje-McKenna M Parrish CR. Parvovirus family conundrum: what makes a killer? Annu Rev Virol. (2015) 2:425–50. doi: 10.1146/annurev-virology-100114-055150, PMID: 26958923

[8] Mietzsch M Penzes JJ Agbandje-McKenna M. Twenty-five years of structural Parvovirology. Viruses. (2019) 11:362. doi: 10.3390/v11040362, PMID: 31010002 PMC6521121

[9] Afumba R Liu JT Dong H. Apoptosis mechanisms induced by parvovirus infections. Acta Virol. (2022) 66:101–9. doi: 10.4149/av_2022_210, PMID: 35766467

[10] Li L Liu Z Liang R Yang M Yan Y Jiao Y. Novel mutation N588 residue in the NS1 protein of feline parvovirus greatly augments viral replication. J Virol. (2024) 98:e0009324. doi: 10.1128/jvi.00093-24, PMID: 38591899 PMC11092363

[11] Mattola S Salokas K Aho V Mantyla E Salminen S Hakanen S. Parvovirus nonstructural protein 2 interacts with chromatin-regulating cellular proteins. PLoS Pathog. (2022) 18:e1010353. doi: 10.1371/journal.ppat.1010353, PMID: 35395063 PMC9020740

[12] Yan YQ Jin LB Wang Y Lu SY Pei YF Zhu DW. Goose parvovirus and the protein NS1 induce apoptosis through the AIF-mitochondrial pathway in goose embryo fibroblasts. Res Vet Sci. (2021) 137:68–76. doi: 10.1016/j.rvsc.2021.04.018, PMID: 33933710

[13] Xu P Zhou Z Xiong M Zou W Deng X Ganaie SS. Parvovirus B19 NS1 protein induces cell cycle arrest at G2-phase by activating the ATR-CDC25C-CDK1 pathway. PLoS Pathog. (2017) 13:e1006266. doi: 10.1371/journal.ppat.1006266, PMID: 28264028 PMC5354443

[14] Gupta SK Sahoo AP Rosh N Gandham RK Saxena L Singh AK. Canine parvovirus NS1 induced apoptosis involves mitochondria, accumulation of reactive oxygen species and activation of caspases. Virus Res. (2016) 213:46–61. doi: 10.1016/j.virusres.2015.10.019, PMID: 26555166

[15] Zhang S Yang J Wang Z Chen L Diao Y Tang Y. Research note: development of an ELISA to distinguish between goose parvovirus infection and vaccine immunization antibodies. Poult Sci. (2020) 99:1332–40. doi: 10.1016/j.psj.2019.12.012, PMID: 32111309 PMC7587739

[16] Yang YT Deng ZC Zhang LJ Fu XL Fu C Zhan XZ. Novel goose parvovirus VP1 targets IRF7 protein to block the type I interferon upstream signaling pathway. Poult Sci. (2024) 103:104065. doi: 10.1016/j.psj.2024.104065, PMID: 39043024 PMC11318561

[17] Callaway HM Feng KH Lee DW Allison AB Pinard M McKenna R. Parvovirus capsid structures required for infection: mutations controlling receptor recognition and protease cleavages. J Virol. (2017) 91:e01871-16. doi: 10.1128/JVI.01871-16, PMID: 27847360 PMC5215354

[18] Zhuandi G Zhaofang Y Dianyu L Mengyuan P Suocheng W. Immune escape of bovine parvovirus by VP1 inhibiting IFN-beta production through the RIG-I-like receptor pathway. Int Microbiol. (2023) 26:757–64. doi: 10.1007/s10123-023-00330-8, PMID: 36703013 PMC9879738

[19] Lopez-Astacio RA Adu OF Goetschius DJ Lee H Weichert WS Wasik BR. Viral capsid, antibody, and receptor interactions: experimental analysis of the antibody escape evolution of canine parvovirus. J Virol. (2023) 97:e0009023. doi: 10.1128/jvi.00090-23, PMID: 37199627 PMC10308881

[20] Asiimwe R Alexander D. STAR+WASP reduces reference bias in the allele-specific mapping of RNA-seq reads. bio Rxiv. (2024) 576391. doi: 10.1101/2024.01.21.576391, PMID: 38370773 PMC10871176

[21] Li Y Gao G Lin Y Hu S Luo Y Wang G. Pacific biosciences assembly with hi-C mapping generates an improved, chromosome-level goose genome. Gigascience. (2020) 9:giaa114. doi: 10.1093/gigascience/giaa114, PMID: 33099628 PMC7585555

[22] Cao P Sun Z Zhang F Zhang J Zheng X Yu B. TGF-beta enhances immunosuppression of myeloid-derived suppressor cells to induce transplant immune tolerance through affecting Arg-1 expression. Front Immunol. (2022) 13:919674. doi: 10.3389/fimmu.2022.919674, PMID: 35874674 PMC9300822

[23] Liu S Li J Zhang Y Wang C Zhang L. IL-10: the master immunomodulatory cytokine in allergen immunotherapy. Expert Rev Clin Immunol. (2024) 21:17–28. doi: 10.1080/1744666X.2024.2406894, PMID: 39323099

[24] He K Xiao H Mac Donald WA Mehta I Kishore A Vincent A. Spatial microniches of IL-2 combine with IL-10 to drive lung migratory T (H)2 cells in response to inhaled allergen. Nat Immunol. (2024) 25:2124–39. doi: 10.1038/s41590-024-01986-8, PMID: 39394532 PMC11934206

[25] Kapgate SS Kumanan K Vijayarani K Barbuddhe SB. Avian parvovirus: classification, phylogeny, pathogenesis and diagnosis. Avian Pathol. (2018) 47:536–45. doi: 10.1080/03079457.2018.1517938, PMID: 30246559

[26] Mattola S Hakanen S Salminen S Aho V Mantyla E Ihalainen TO. Concepts to reveal parvovirus-nucleus interactions. Viruses. (2021) 13:1306. doi: 10.3390/v13071306, PMID: 34372512 PMC8310053

[27] Chen DY Tzang CC Liu CM Chiu TM Lin JW Chuang PH. Effect of the functional VP1 unique region of human parvovirus B19 in causing skin fibrosis of systemic sclerosis. Int J Mol Sci. (2023) 24:15294. doi: 10.3390/ijms24201529 37894973 PMC10607574

[28] Mitchell LA. Parvovirus B19 nonstructural (NS1) protein as a transactivator of interleukin-6 synthesis: common pathway in inflammatory sequelae of human parvovirus infections? J Med Virol. (2002) 67:267–74. doi: 10.1002/jmv.2217, PMID: 11992589

[29] De Benedetti F Rucci N Del Fattore A Peruzzi B Paro R Longo M. Impaired skeletal development in interleukin-6-transgenic mice: a model for the impact of chronic inflammation on the growing skeletal system. Arthritis Rheum. (2006) 54:3551–63. doi: 10.1002/art.22175, PMID: 17075861

[30] Maugeri D Mamazza C Lo Giudice F Puglisi N Muscoso EG Rizzotto M. Interleukin-18 (IL-18) and matrix metalloproteinase-9 (MMP-9) in post-menopausal osteoporosis. Arch Gerontol Geriatr. (2005) 40:299–305. doi: 10.1016/j.archger.2004.10.001, PMID: 15814163

[31] Da W Jiang W Tao L. ROS/MMP-9 mediated CS degradation in BMSC inhibits citric acid metabolism participating in the dual regulation of bone remodelling. Cell Death Dis. (2024) 10:77. doi: 10.1038/s41420-024-01835-5, PMID: 38355572 PMC10866869

[32] Hou Y Huttenlocher A. Advancing chemokine research: the molecular function of CXCL8. J Clin Invest. (2024) 134:984. doi: 10.1172/JCI180984, PMID: 38747289 PMC11093595

[33] Greenhill C. CCN3 maintains bone density during lactation. Nat Rev Endocrinol. (2024) 20:507. doi: 10.1038/s41574-024-01023-6, PMID: 39043957

[34] de OCP GFA Figueiredo LB Zaramela LS ESA P Godinho RO Gomes MD. Identification of potential target genes associated with the reversion of androgen-dependent skeletal muscle atrophy. Arch Biochem Biophys. (2019) 663:173–82. doi: 10.1016/j.abb.2019.01.009, PMID: 30639329

[35] Kosla J Dvorak M Cermak V. Molecular analysis of the TGF-beta controlled gene expression program in chicken embryo dermal myofibroblasts. Gene. (2013) 513:90–100. doi: 10.1016/j.gene.2012.10.069, PMID: 23127594

[36] Wang J Zhao X Wan YY. Intricacies of TGF-beta signaling in Treg and Th17 cell biology. Cell Mol Immunol. (2023) 20:1002–22. doi: 10.1038/s41423-023-01036-7, PMID: 37217798 PMC10468540

[37] Gallinella G. New insights into parvovirus research. Viruses. (2019) 11:1053. doi: 10.3390/v11111053, PMID: 31766142 PMC6893750

[38] Xu M Leskinen K Gritti T Groma V Arola J Lepisto A. Prevalence, cell tropism, and clinical impact of human parvovirus persistence in adenomatous, cancerous, inflamed, and healthy intestinal mucosa. Front Microbiol. (2022) 13:914181. doi: 10.3389/fmicb.2022.914181, PMID: 35685923 PMC9171052

[39] Majumder K Boftsi M Whittle FB Wang J Fuller MS Joshi T. The NS1 protein of the parvovirus MVM aids in the localization of the viral genome to cellular sites of DNA damage. PLoS Pathog. (2020) 16:e1009002. doi: 10.1371/journal.ppat.1009002, PMID: 33064772 PMC7592911

[40] Dai X Zhang X Miao Y Han P Zhang J. Canine parvovirus induces G1/S cell cycle arrest that involves EGFR Tyr1086 phosphorylation. Virulence. (2020) 11:1203–14. doi: 10.1080/21505594.2020.1814091, PMID: 32877289 PMC7549965

[41] Ducloux C You B Langele A Goupille O Payen E Chretien S. Enhanced cell-based detection of parvovirus B19V infectious units according to cell cycle status. Viruses. (2020) 12:1467. doi: 10.3390/v12121467, PMID: 33353185 PMC7766612

[42] Miao B Chen S Zhang X Ma P Ma M Chen C. T598 and T601 phosphorylation sites of canine parvovirus NS1 are crucial for viral replication and pathogenicity. Vet Microbiol. (2022) 264:109301. doi: 10.1016/j.vetmic.2021.109301 34915313

[43] Kwak SH Kim H Yun H Lim J Kang DH Kim D. Characterization of natural compounds as inhibitors of NS1 endonuclease from canine parvovirus type 2. J Microbiol Biotechnol. (2023) 33:788–96. doi: 10.4014/jmb.2211.11040, PMID: 36994623 PMC10331946

[44] Magerus A Bercher-Brayer C Rieux-Laucat F. The genetic landscape of the FAS pathway deficiencies. Biom J. (2021) 44:388–99. doi: 10.1016/j.bj.2021.06.005, PMID: 34171534 PMC8514852

[45] Ji W Xin Y Zhang L Liu X. ALG2 influences T cell apoptosis by regulating FASLG intracellular transportation. Biochem J. (2020) 477:3105–21. doi: 10.1042/BCJ20200028, PMID: 32766719

[46] Wang Y Chen G Shao W. Identification of Ferroptosis-related genes in Alzheimer's disease based on Bioinformatic analysis. Front Neurosci. (2022) 16:823741. doi: 10.3389/fnins.2022.823741, PMID: 35197821 PMC8858973

[47] Xu D Zhou C Lin J Cai W Lin W. MicroRNA-367-3p suppresses sevoflurane-induced adult rat astrocyte apoptosis by targeting BCL2L11. Exp Ther Med. (2022) 23:9. doi: 10.3892/etm.2021.10931, PMID: 34815761 PMC8593860

[48] Tang D Chen X Kang R Kroemer G. Ferroptosis: molecular mechanisms and health implications. Cell Res. (2021) 31:107–25. doi: 10.1038/s41422-020-00441-1, PMID: 33268902 PMC8026611

[49] Xu XQ Xu T Ji W Wang C Ren Y Xiong X. Herpes simplex virus 1-induced Ferroptosis contributes to viral encephalitis. MBio. (2023) 14:e0237022. doi: 10.1128/mbio.02370-22, PMID: 36507835 PMC9973258

[50] Giansanti F Rossi P Massucci MT Botti D Antonini G Valenti P. Antiviral activity of ovotransferrin discloses an evolutionary strategy for the defensive activities of lactoferrin. Biochem Cell Biol. (2002) 80:125–30. doi: 10.1139/o01-208, PMID: 11908636

[51] Beatty A Singh T Tyurina YY Tyurin VA Samovich S Nicolas E. Ferroptotic cell death triggered by conjugated linolenic acids is mediated by ACSL1. Nat Commun. (2021) 12:2244. doi: 10.1038/s41467-021-22471-y, PMID: 33854057 PMC8046803

