# Supplementary material for: Insights into growth retardation and dwarfism caused by goose parvovirus in goslings: a transcriptomic profiling study
Source: Front Vet Sci. 2025 May 6;12:1529978. doi: 10.3389/fvets.2025.1529978 (PMC12090931; doi:10.3389/fvets.2025.1529978)
Supplement: Supplementary file 1 [file Image_1.pdf]

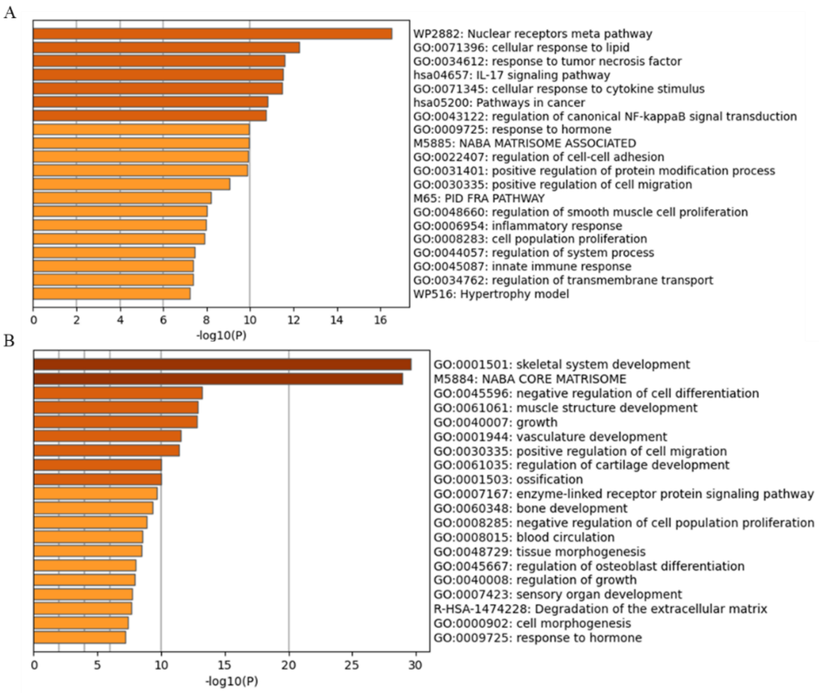

Figure S1 The functional enrichment analysis of up-regulated and down-regulated DEGs caused by GPV infection on GEFs. (A) The functional enrichment analysis of up-regulated DEGs, (B) The functional enrichment analysis of down-regulated DEGs.
