# Supplementary material for: Insights into growth retardation and dwarfism caused by goose parvovirus in goslings: a transcriptomic profiling study
Source: Front Vet Sci. 2025 May 6;12:1529978. doi: 10.3389/fvets.2025.1529978 (PMC12090931; doi:10.3389/fvets.2025.1529978)
Supplement: Supplementary file 2 [file Table_1.docx]

| Table S1. The primer sequences of selected candidate genes in RT–qPCR | | |
| --- | --- | --- |
| Gene | NCBI accession identifier | Sequence |
| *GAPDH* | XM_013199522 | GTGGTGCTAAGCGTGTCA, GGCTGGGATAATGTTCTGG |
| *LTF* | XM_013186329 | ACCAACTACTACGCTGTGGC, GCCTGAGTCTATGCCTTCCC |
| CXCL8 | XM_013190618 | CAAGGCAGGAGCCTGGTAAG, ACAGCGGTGCATCAGAATTG |
| *PTGS2* | XM_048058960 | GCCAATCCTTGCTGCTCAAAC,TGGCGTAGGTTTCAATGTCAGT |
| *MMP9* | XM_048050603 | TTTGGCTACACCACAGAGGC, CTATCACAGCACGGTCCAGG |
| *PDGFB* | XM_048069820 | GATCCCTGACAGCCACAACA, CGTAGGTCCAGTGCAAGTGT |
| *MMP2* | XM_013192370 | GGAGAGGGGCAAGTGGTTAG, AACACCAGAGGAACCCATCG |
| *ACSL1* | XM_048078356 | CTGGTATGCGACAAGACCGA, TGGAGTAATGCAGAGCCCAC |
| *EXFAB* | XM_013182731 | CCAGCCAGGATGAATGCAGA, AGCCCTGAAGAGGGATAGCA |
| *CCN3* | XM_066990693 | GTGCTGCGAGAAGTGGATCT, CAAAGGCGTGTCTGCTTCAC |
| *FAM180A* | XM_013173073 | GGACACATGTAGCACGTCCA, AACATGCCTCTAAGCCAGCA |
| *PI15* | XM_013172440 | GCGATGCTATGGACCCATGT, CCAAGTAAACAGCTCGTCGC |
